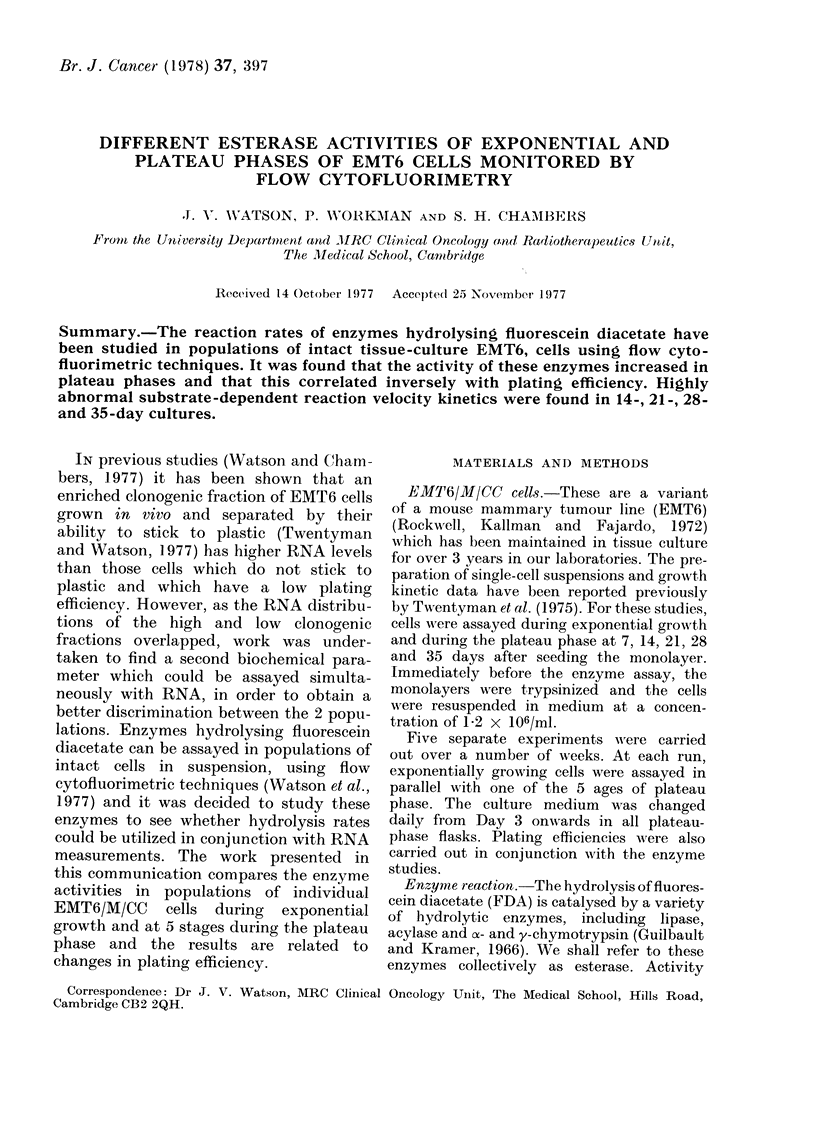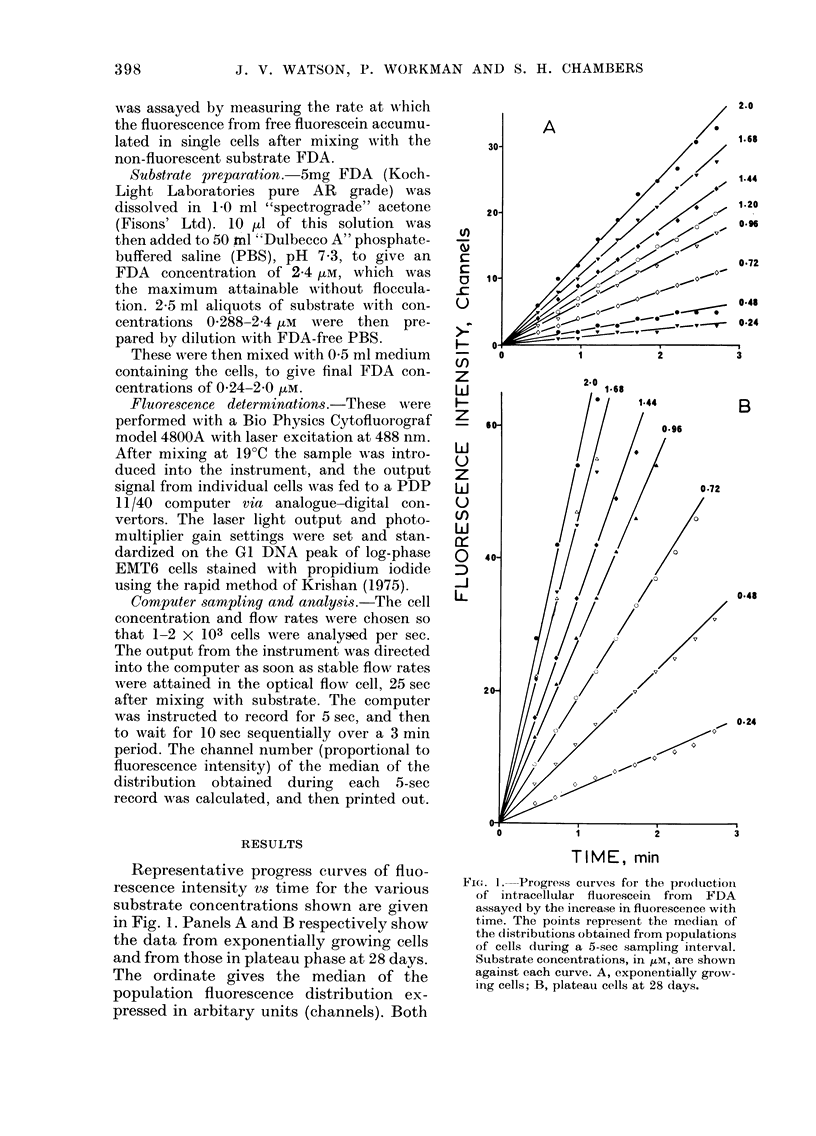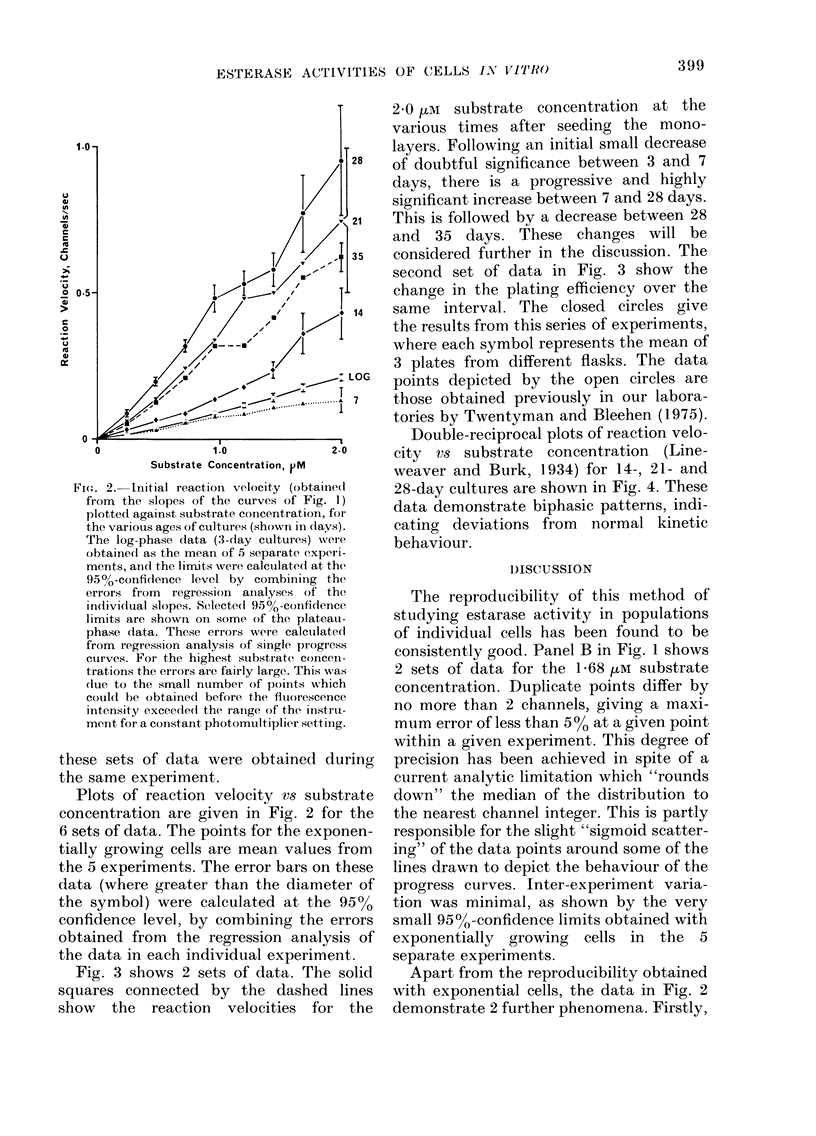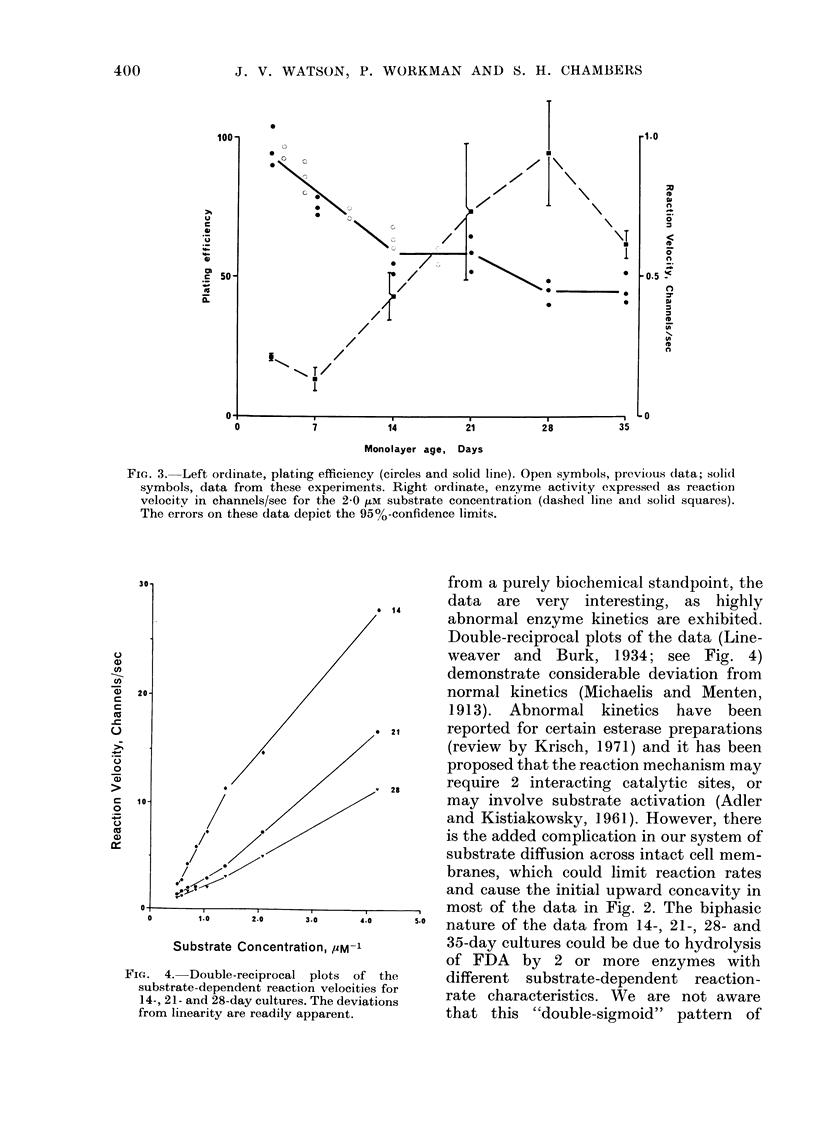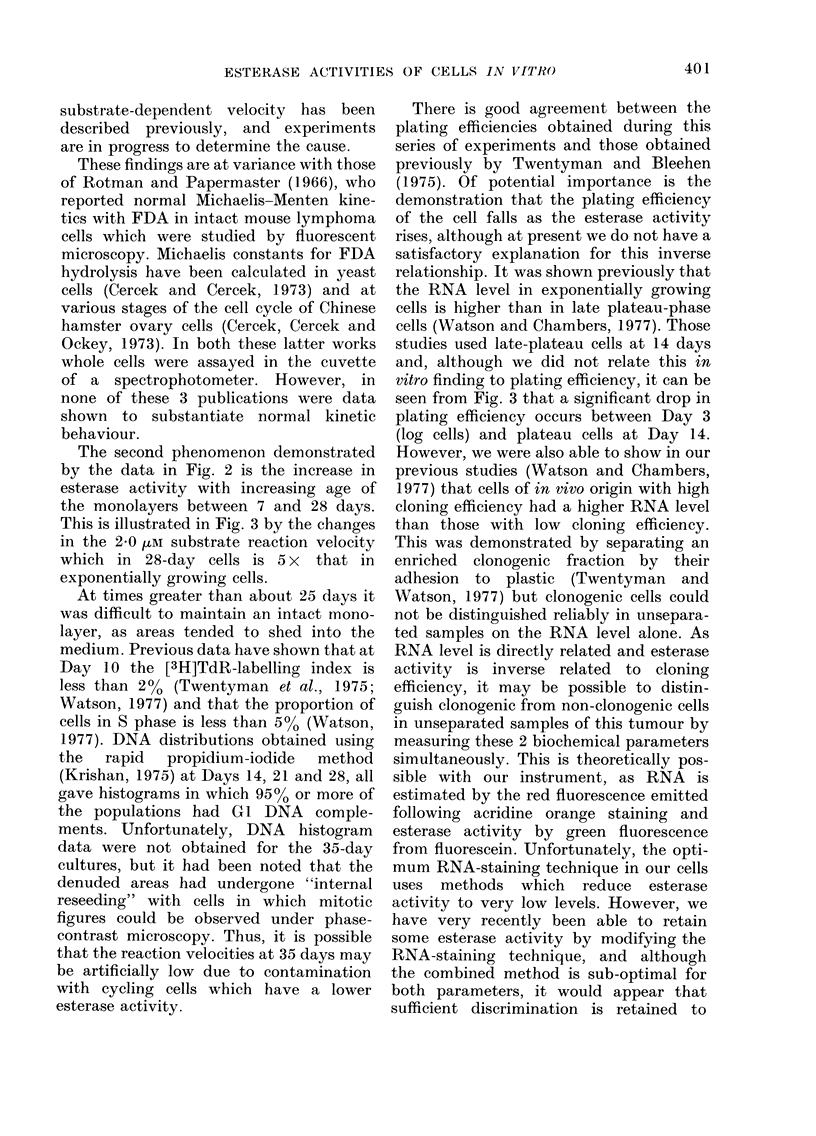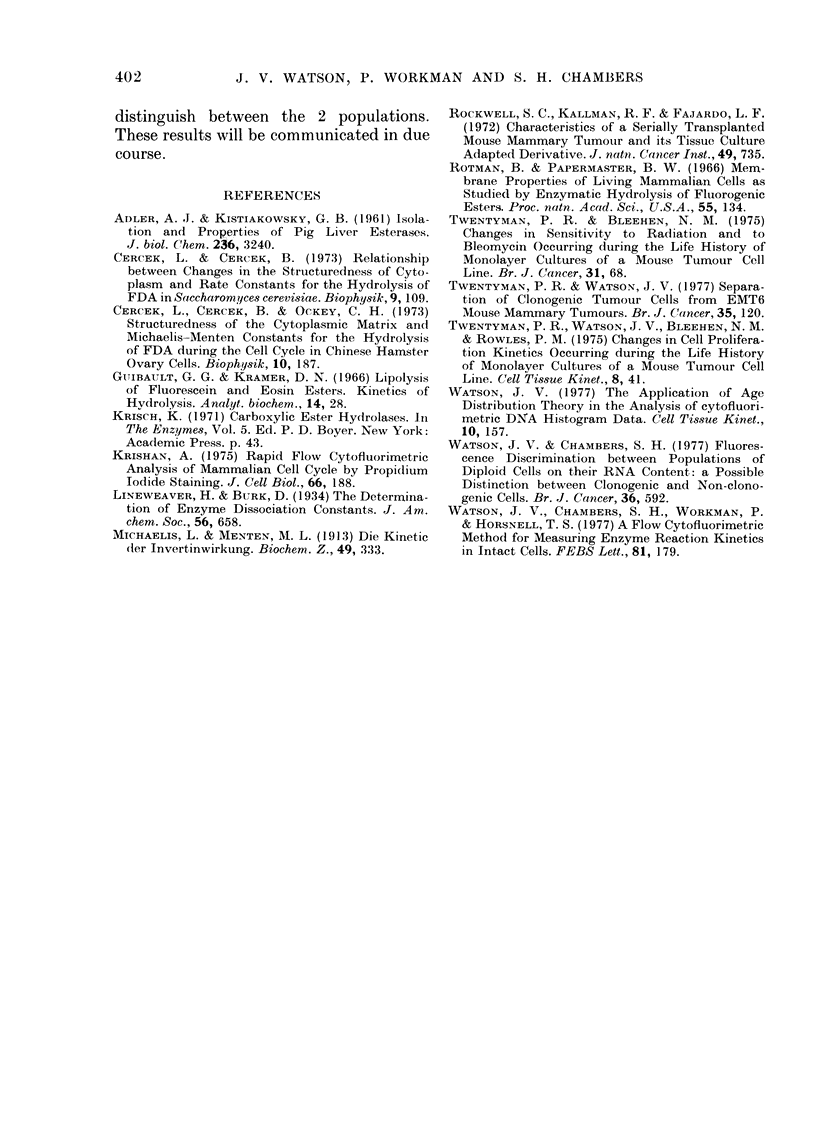# Different esterase activities of exponential and plateau phases of EMT6 cells monitored by flow cytofluorimetry.

**DOI:** 10.1038/bjc.1978.59

**Published:** 1978-03

**Authors:** J. V. Watson, P. Workman, S. H. Chambers

## Abstract

The reaction rates of enzymes hydrolysing fluorescein diacetate have been studied in populations of intact tissue-culture EMT6, cells using flow cytofluorimetric techniques. It was found that the activity of these enzymes increased in plateau phases and that this correlated inversely with plating efficiency. Highly abnormal substrate-dependent reaction velocity kinetics were found in 14-, 21-, 28- and 35-day cultures.


*Br. J. Cancer* (1978) **37**, 397

### DIFFERENT ESTERASE ACTIVITIES OF EXPONENTIAL AND PLATEAU PHASES OF EMT6 CELLS MONITORED BY FLOW CYTOFLUORIMETRY

J. V. WATSON, P. WORKMAN AND S. H. CHAMBERS

*From the University Department and MRC Clinical Oncology and Radiotherapeutics Unit, The Medical School, Cambridge*

Received 14 October 1977 Accepted 25 November 1977

**Summary.—The reaction rates of enzymes hydrolysing fluorescein diacetate have been studied in populations of intact tissue-culture EMT6, cells using flow cytofluorimetric techniques. It was found that the activity of these enzymes increased in plateau phases and that this correlated inversely with plating efficiency. Highly abnormal substrate-dependent reaction velocity kinetics were found in 14-, 21-, 28- and 35-day cultures.**

IN previous studies (Watson and Chambers, 1977) it has been shown that an enriched clonogenic fraction of EMT6 cells grown *in vivo* and separated by their ability to stick to plastic (Twentyman and Watson, 1977) has higher RNA levels than those cells which do not stick to plastic and which have a low plating efficiency. However, as the RNA distributions of the high and low clonogenic fractions overlapped, work was undertaken to find a second biochemical parameter which could be assayed simultaneously with RNA, in order to obtain a better discrimination between the 2 populations. Enzymes hydrolysing fluorescein diacetate can be assayed in populations of intact cells in suspension, using flow cytofluorimetric techniques (Watson *et al.*, 1977) and it was decided to study these enzymes to see whether hydrolysis rates could be utilized in conjunction with RNA measurements. The work presented in this communication compares the enzyme activities in populations of individual EMT6/M/CC cells during exponential growth and at 5 stages during the plateau phase and the results are related to changes in plating efficiency.

### MATERIALS AND METHODS

*EMT6/M/CC cells.*—These are a variant of a mouse mammary tumour line (EMT6) (Rockwell, Kallman and Fajardo, 1972) which has been maintained in tissue culture for over 3 years in our laboratories. The preparation of single-cell suspensions and growth kinetic data have been reported previously by Twentyman *et al.* (1975). For these studies, cells were assayed during exponential growth and during the plateau phase at 7, 14, 21, 28 and 35 days after seeding the monolayer. Immediately before the enzyme assay, the monolayers were trypsinized and the cells were resuspended in medium at a concentration of $1{\cdot}2 \times 10^6$/ml.

Five separate experiments were carried out over a number of weeks. At each run, exponentially growing cells were assayed in parallel with one of the 5 ages of plateau phase. The culture medium was changed daily from Day 3 onwards in all plateau-phase flasks. Plating efficiencies were also carried out in conjunction with the enzyme studies.

*Enzyme reaction.*—The hydrolysis of fluorescein diacetate (FDA) is catalysed by a variety of hydrolytic enzymes, including lipase, acylase and $\alpha$- and $\gamma$-chymotrypsin (Guilbault and Kramer, 1966). We shall refer to these enzymes collectively as esterase. Activity

Correspondence: Dr J. V. Watson, MRC Clinical Oncology Unit, The Medical School, Hills Road, Cambridge CB2 2QH.

was assayed by measuring the rate at which the fluorescence from free fluorescein accumulated in single cells after mixing with the non-fluorescent substrate FDA.

*Substrate preparation.*—5mg FDA (Koch-Light Laboratories pure AR grade) was dissolved in 1·0 ml "spectrograde" acetone (Fisons' Ltd). 10 $\mu$l of this solution was then added to 50 ml "Dulbecco A" phosphate-buffered saline (PBS), pH 7·3, to give an FDA concentration of 2·4 $\mu$M, which was the maximum attainable without flocculation. 2·5 ml aliquots of substrate with concentrations 0·288–2·4 $\mu$M were then prepared by dilution with FDA-free PBS.

These were then mixed with 0·5 ml medium containing the cells, to give final FDA concentrations of 0·24–2·0 $\mu$M.

*Fluorescence determinations.*—These were performed with a Bio Physics Cytofluorograf model 4800A with laser excitation at 488 nm. After mixing at 19°C the sample was introduced into the instrument, and the output signal from individual cells was fed to a PDP 11/40 computer *via* analogue–digital convertors. The laser light output and photomultiplier gain settings were set and standardized on the G1 DNA peak of log-phase EMT6 cells stained with propidium iodide using the rapid method of Krishan (1975).

*Computer sampling and analysis.*—The cell concentration and flow rates were chosen so that 1–2 × $10^3$ cells were analysed per sec. The output from the instrument was directed into the computer as soon as stable flow rates were attained in the optical flow cell, 25 sec after mixing with substrate. The computer was instructed to record for 5 sec, and then to wait for 10 sec sequentially over a 3 min period. The channel number (proportional to fluorescence intensity) of the median of the distribution obtained during each 5-sec record was calculated, and then printed out.

FIG. 1.—Progress curves for the production of intracellular fluorescein from FDA assayed by the increase in fluorescence with time. The points represent the median of the distributions obtained from populations of cells during a 5-sec sampling interval. Substrate concentrations, in $\mu$M, are shown against each curve. A, exponentially growing cells; B, plateau cells at 28 days.

### RESULTS

Representative progress curves of fluorescence intensity *vs* time for the various substrate concentrations shown are given in Fig. 1. Panels A and B respectively show the data from exponentially growing cells and from those in plateau phase at 28 days. The ordinate gives the median of the population fluorescence distribution expressed in arbitary units (channels). Both

FIG. 2.—Initial reaction velocity (obtained from the slopes of the curves of Fig. 1) plotted against substrate concentration, for the various ages of cultures (shown in days). The log-phase data (3-day cultures) were obtained as the mean of 5 separate experiments, and the limits were calculated at the 95%-confidence level by combining the errors from regression analyses of the individual slopes. Selected 95%-confidence limits are shown on some of the plateau-phase data. These errors were calculated from regression analysis of single progress curves. For the highest substrate concentrations the errors are fairly large. This was due to the small number of points which could be obtained before the fluorescence intensity exceeded the range of the instrument for a constant photomultiplier setting.

these sets of data were obtained during the same experiment.

Plots of reaction velocity *vs* substrate concentration are given in Fig. 2 for the 6 sets of data. The points for the exponentially growing cells are mean values from the 5 experiments. The error bars on these data (where greater than the diameter of the symbol) were calculated at the 95% confidence level, by combining the errors obtained from the regression analysis of the data in each individual experiment.

Fig. 3 shows 2 sets of data. The solid squares connected by the dashed lines show the reaction velocities for the 2·0 µM substrate concentration at the various times after seeding the monolayers. Following an initial small decrease of doubtful significance between 3 and 7 days, there is a progressive and highly significant increase between 7 and 28 days. This is followed by a decrease between 28 and 35 days. These changes will be considered further in the discussion. The second set of data in Fig. 3 show the change in the plating efficiency over the same interval. The closed circles give the results from this series of experiments, where each symbol represents the mean of 3 plates from different flasks. The data points depicted by the open circles are those obtained previously in our laboratories by Twentyman and Bleehen (1975).

Double-reciprocal plots of reaction velocity *vs* substrate concentration (Lineweaver and Burk, 1934) for 14-, 21- and 28-day cultures are shown in Fig. 4. These data demonstrate biphasic patterns, indicating deviations from normal kinetic behaviour.

### DISCUSSION

The reproducibility of this method of studying estarase activity in populations of individual cells has been found to be consistently good. Panel B in Fig. 1 shows 2 sets of data for the 1·68 µM substrate concentration. Duplicate points differ by no more than 2 channels, giving a maximum error of less than 5% at a given point within a given experiment. This degree of precision has been achieved in spite of a current analytic limitation which "rounds down" the median of the distribution to the nearest channel integer. This is partly responsible for the slight "sigmoid scattering" of the data points around some of the lines drawn to depict the behaviour of the progress curves. Inter-experiment variation was minimal, as shown by the very small 95%-confidence limits obtained with exponentially growing cells in the 5 separate experiments.

Apart from the reproducibility obtained with exponential cells, the data in Fig. 2 demonstrate 2 further phenomena. Firstly,

FIG. 3.—Left ordinate, plating efficiency (circles and solid line). Open symbols, previous data; solid symbols, data from these experiments. Right ordinate, enzyme activity expressed as reaction velocity in channels/sec for the 2·0 $\mu$M substrate concentration (dashed line and solid squares). The errors on these data depict the 95%-confidence limits.

FIG. 4.—Double-reciprocal plots of the substrate-dependent reaction velocities for 14-, 21- and 28-day cultures. The deviations from linearity are readily apparent.

from a purely biochemical standpoint, the data are very interesting, as highly abnormal enzyme kinetics are exhibited. Double-reciprocal plots of the data (Lineweaver and Burk, 1934; see Fig. 4) demonstrate considerable deviation from normal kinetics (Michaelis and Menten, 1913). Abnormal kinetics have been reported for certain esterase preparations (review by Krisch, 1971) and it has been proposed that the reaction mechanism may require 2 interacting catalytic sites, or may involve substrate activation (Adler and Kistiakowsky, 1961). However, there is the added complication in our system of substrate diffusion across intact cell membranes, which could limit reaction rates and cause the initial upward concavity in most of the data in Fig. 2. The biphasic nature of the data from 14-, 21-, 28- and 35-day cultures could be due to hydrolysis of FDA by 2 or more enzymes with different substrate-dependent reaction-rate characteristics. We are not aware that this "double-sigmoid" pattern of substrate-dependent velocity has been described previously, and experiments are in progress to determine the cause.

These findings are at variance with those of Rotman and Papermaster (1966), who reported normal Michaelis–Menten kinetics with FDA in intact mouse lymphoma cells which were studied by fluorescent microscopy. Michaelis constants for FDA hydrolysis have been calculated in yeast cells (Cercek and Cercek, 1973) and at various stages of the cell cycle of Chinese hamster ovary cells (Cercek, Cercek and Ockey, 1973). In both these latter works whole cells were assayed in the cuvette of a spectrophotometer. However, in none of these 3 publications were data shown to substantiate normal kinetic behaviour.

The second phenomenon demonstrated by the data in Fig. 2 is the increase in esterase activity with increasing age of the monolayers between 7 and 28 days. This is illustrated in Fig. 3 by the changes in the 2·0 $\mu$M substrate reaction velocity which in 28-day cells is 5× that in exponentially growing cells.

At times greater than about 25 days it was difficult to maintain an intact monolayer, as areas tended to shed into the medium. Previous data have shown that at Day 10 the [$^3$H]TdR-labelling index is less than 2% (Twentyman *et al.*, 1975; Watson, 1977) and that the proportion of cells in S phase is less than 5% (Watson, 1977). DNA distributions obtained using the rapid propidium-iodide method (Krishan, 1975) at Days 14, 21 and 28, all gave histograms in which 95% or more of the populations had G1 DNA complements. Unfortunately, DNA histogram data were not obtained for the 35-day cultures, but it had been noted that the denuded areas had undergone "internal reseeding" with cells in which mitotic figures could be observed under phase-contrast microscopy. Thus, it is possible that the reaction velocities at 35 days may be artificially low due to contamination with cycling cells which have a lower esterase activity.

There is good agreement between the plating efficiencies obtained during this series of experiments and those obtained previously by Twentyman and Bleehen (1975). Of potential importance is the demonstration that the plating efficiency of the cell falls as the esterase activity rises, although at present we do not have a satisfactory explanation for this inverse relationship. It was shown previously that the RNA level in exponentially growing cells is higher than in late plateau-phase cells (Watson and Chambers, 1977). Those studies used late-plateau cells at 14 days and, although we did not relate this *in vitro* finding to plating efficiency, it can be seen from Fig. 3 that a significant drop in plating efficiency occurs between Day 3 (log cells) and plateau cells at Day 14. However, we were also able to show in our previous studies (Watson and Chambers, 1977) that cells of *in vivo* origin with high cloning efficiency had a higher RNA level than those with low cloning efficiency. This was demonstrated by separating an enriched clonogenic fraction by their adhesion to plastic (Twentyman and Watson, 1977) but clonogenic cells could not be distinguished reliably in unseparated samples on the RNA level alone. As RNA level is directly related and esterase activity is inverse related to cloning efficiency, it may be possible to distinguish clonogenic from non-clonogenic cells in unseparated samples of this tumour by measuring these 2 biochemical parameters simultaneously. This is theoretically possible with our instrument, as RNA is estimated by the red fluorescence emitted following acridine orange staining and esterase activity by green fluorescence from fluorescein. Unfortunately, the optimum RNA-staining technique in our cells uses methods which reduce esterase activity to very low levels. However, we have very recently been able to retain some esterase activity by modifying the RNA-staining technique, and although the combined method is sub-optimal for both parameters, it would appear that sufficient discrimination is retained to distinguish between the 2 populations. These results will be communicated in due course.

### REFERENCES

ADLER, A. J. & KISTIAKOWSKY, G. B. (1961) Isolation and Properties of Pig Liver Esterases. *J. biol. Chem.* **236**, 3240.

CERCEK, L. & CERCEK, B. (1973) Relationship between Changes in the Structuredness of Cytoplasm and Rate Constants for the Hydrolysis of FDA in *Saccharomyces cerevisiae. Biophysik*, **9**, 109.

CERCEK, L., CERCEK, B. & OCKEY, C. H. (1973) Structuredness of the Cytoplasmic Matrix and Michaelis-Menten Constants for the Hydrolysis of FDA during the Cell Cycle in Chinese Hamster Ovary Cells. *Biophysik*, **10**, 187.

GUIBAULT, G. G. & KRAMER, D. N. (1966) Lipolysis of Fluorescein and Eosin Esters. Kinetics of Hydrolysis. *Analyt. biochem.*, **14**, 28.

KRISCH, K. (1971) Carboxylic Ester Hydrolases. In *The Enzymes*, Vol. 5. Ed. P. D. Boyer. New York: Academic Press. p. 43.

KRISHAN, A. (1975) Rapid Flow Cytofluorimetric Analysis of Mammalian Cell Cycle by Propidium Iodide Staining. *J. Cell Biol.*, **66**, 188.

LINEWEAVER, H. & BURK, D. (1934) The Determination of Enzyme Dissociation Constants. *J. Am. chem. Soc.*, **56**, 658.

MICHAELIS, L. & MENTEN, M. L. (1913) Die Kinetic der Invertinwirkung. *Biochem. Z.*, **49**, 333.

ROCKWELL, S. C., KALLMAN, R. F. & FAJARDO, L. F. (1972) Characteristics of a Serially Transplanted Mouse Mammary Tumour and its Tissue Culture Adapted Derivative. *J. natn. Cancer Inst.*, **49**, 735.

ROTMAN, B. & PAPERMASTER, B. W. (1966) Membrane Properties of Living Mammalian Cells as Studied by Enzymatic Hydrolysis of Fluorogenic Esters. *Proc. natn. Acad. Sci., U.S.A.*, **55**, 134.

TWENTYMAN, P. R. & BLEEHEN, N. M. (1975) Changes in Sensitivity to Radiation and to Bleomycin Occurring during the Life History of Monolayer Cultures of a Mouse Tumour Cell Line. *Br. J. Cancer*, **31**, 68.

TWENTYMAN, P. R. & WATSON, J. V. (1977) Separation of Clonogenic Tumour Cells from EMT6 Mouse Mammary Tumours. *Br. J. Cancer*, **35**, 120.

TWENTYMAN, P. R., WATSON, J. V., BLEEHEN, N. M. & ROWLES, P. M. (1975) Changes in Cell Proliferation Kinetics Occurring during the Life History of Monolayer Cultures of a Mouse Tumour Cell Line. *Cell Tissue Kinet.*, **8**, 41.

WATSON, J. V. (1977) The Application of Age Distribution Theory in the Analysis of cytofluorimetric DNA Histogram Data. *Cell Tissue Kinet.*, **10**, 157.

WATSON, J. V. & CHAMBERS, S. H. (1977) Fluorescence Discrimination between Populations of Diploid Cells on their RNA Content: a Possible Distinction between Clonogenic and Non-clonogenic Cells. *Br. J. Cancer*, **36**, 592.

WATSON, J. V., CHAMBERS, S. H., WORKMAN, P. & HORSNELL, T. S. (1977) A Flow Cytofluorimetric Method for Measuring Enzyme Reaction Kinetics in Intact Cells. *FEBS Lett.*, **81**, 179.